# Enhancing encoding through repeated study affects retrieval related pupil dilation during cued recall, but not during recognition

**DOI:** 10.1038/s41598-026-40350-8

**Published:** 2026-02-17

**Authors:** Ádám Albi, Péter Pajkossy

**Affiliations:** Department of Cognitive Science, Faculty of Natural Sciences, Budapest University of Technology and Economics, Műegyetem rkp. 3, Budapest, H-1111 Hungary

**Keywords:** Episodic memory, Pupil dilation, Memory trace strength, Mental effort, Pupil old/new effect, Neuroscience, Physiology, Psychology

## Abstract

In addition to its well-known association with mental effort, pupil dilation provides insight into the cognitive and neurobiological basis of episodic memory. Notably, pupil responses during retrieval are influenced by the encoding conditions under which the information was originally learned. In our study, we manipulated encoding by presenting participants with word pairs either once or twice, and then examined pupil responses during subsequent retrieval tasks that employed either a recognition or a cued recall paradigm. Repeated encoding led to higher accuracy and faster response times, confirming enhanced memory performance across both tasks. Critically, however, the strengthening of memory traces was accompanied by reduced pupil dilation only in the cued recall task, suggesting that stronger memories required less cognitive effort during the demanding recall process. In contrast, during the recognition task, despite a robust pupil old/new effect (e.g. increased pupil responses to correct “old” responses relative to correct “new” responses) the magnitude of pupil dilation was not modulated by memory trace strength. This implies that, in recognition decisions, pupil responses may primarily reflect the recollection–familiarity dichotomy rather than memory trace strength. Overall, our findings demonstrate that the type of memory test influences how effectively pupillometry can assess memory trace strength.

**Supplementary Information:**

The online version contains supplementary material available at 10.1038/s41598-026-40350-8.

Episodic memory is an important cognitive process that enables us to recall the details of an earlier experience and it also plays an important role in how new information is acquired and retained^1–4^. Some of our experiences are easily retrieved with high confidence and detail, whereas other events are recalled improperly or with low confidence. Importantly, dynamics at retrieval are significantly determined by encoding conditions: the specifics of the initial exposure inherently determine how we can access the processed information later^5–8^. Thus, differences in encoding conditions significantly influence memory trace strength, that is how well a memory is encoded and consequently, how easily we can retrieve it later. Because of this, the online measurement of memory trace strength is of utmost theoretical and practical importance, for example in educational context.

One promising method for online assessment of memory trace strength might be the measurement of small, non-luminance mediated changes in the size of the pupil. The relationship between mental effort and pupil dilation (PD) has long been established^9,10^, and PD during cognitive processing was proposed as a reflection of general arousal or the summed activity of the brain^11^. Importantly, when luminance related pupil size changes are properly controlled, the pupil dilates more to cognitively demanding tasks^12^, and this links PD to cognitive processing^10,11,13^.

Because of this link, pupillometry might be a promising method to assess how well a given set of information was encoded. A straightforward way to do this would be to measure PD during the encoding of information. Unfortunately, however, PD related to cognitive processing can be best understood as a phasic response triggered by a well-defined cognitive process and characterized by a maximal duration of about a couple of seconds^11,13^. In real-life settings, encoding of information often involves longer time periods and it might recruit a variety of different cognitive processes, which differently affect PD responses, such as novelty processing, and encoding effort^14^. In contrast, the retrieval of information is more restricted regarding both the involved processes and its duration. Because of this, measuring PD during the retrieval of information might be a more promising way to the online assessment of memory trace strength. Interestingly, several studies have found that memory processes may specifically contribute to PD during memory retrieval^15–17^. In one of the earliest studies, Gardner, Mo, and Borrego^18^ presented subjects with previously studied items (i.e. targets) and novel, non-studied items (i.e. foils), and asked them to recognize which item was “old”, that is presented previously. The authors found significantly higher PD when participants correctly recognized targets, as compared to cases when they correctly identified foils. More recently, several studies found a similar pattern showing that correct ‘old’ responses to previously studied target items elicited larger pupil responses compared to correct ‘new’ responses to previously not presented foil items^15,17,19–22^. This effect was termed the pupil old/new effect, and its presence opens up the possibility to investigate the specifics of the retrieval process and the strength of the underlying memory trace using a noninvasive measurement method.

Originally, the pupil old/new effect was interpreted by Vo et al.^22^ as resulting from mental effort, because correct old response to targets (i.e. hits) require the effortful recollection of episodic details which is absent during the correct rejection of novel items. If we assume that better encoded memories—characterized by higher memory trace strength- can be recollected with less effort, than we might predict a negative correlation between memory trace strength and pupil responses during hits: better encoded information should elicit recollection with less mental effort, resulting in smaller PD. In contrast to this, several studies found that successful recognition of target stimuli, which were previously encoded by focusing on the semantic features of the stimuli (deep encoding) elicited larger PD compared to stimuli encoded by focusing on the phonological features (shallow encoding)^21,23^. Because level of processing manipulations results in larger memory trace strength (i.e. better retention)^6^, these results suggest a positive correlation between memory trace strength and PD during recognition memory tasks. Importantly, Taikh and Bodner^23^ showed that the recollection/familiarity dichotomy is an important moderating factor. Dual process models of memory propose that our ability to retrieve memories relies on two distinct mechanisms: one that involves recalling specific contextual details, and another that produces a general sense of familiarity without those details^24–28^. As a result, we might find certain stimuli familiar even when we cannot remember the context in which we encountered them. Using this dichotomy, Taikh and Bodner^23^ showed that recollective responses are associated with larger PD after both shallow and deep encoding, but the proportion of recollective responses is higher for deeply encoded target words, resulting in higher average PD for items after deep, as compared to shallow encoding.

In line with the assumed role of recollection in the pupil old/new effect, several studies showed that hits associated with the recollection of contextual details lead to larger PD than hits relying on a general feeling of stimulus familiarity^19–21^. Thus, whereas the original suggestion of Vo et al.^22^ about the role of recollective recognition in the pupil old/new effect was correct, the results regarding the positive correlation between memory trace strength and PD following hits contradict the suggested causal role of mental effort (which would predict a negative correlation, that is better encoding leads to less effort and thus smaller PD). In contrast, the results of Taikh and Bodner^23^ suggests that the positive link between encoding manipulations and PD during correct hits at subsequent recognition tasks is mediated by the different prevalence of recollective recognition decisions associated with deep vs. shallow encoding. In line with this, recently, Siefert et al.^29^ proposed that the PD during recognition might reflect cue-trace interactions leading to recollective recognition. Thus, we might assume that better encoding might enhance these interactions, leading to the positive correlation between memory trace strength and PD during successful recognition.

In contrast to recognition memory, in the case of cued recall, the link between memory trace strength and PD is better in line with an effort explanation. In this form of retrieval, the previous experience (target) has to be recalled with the help of an associated stimulus, which was encoded together with the target (cue). Specifically, in the case of paired associate learning, a paradigmatic cued recall task, stronger memory traces and thus better memory performances were associated with decreased PD during retrieval^30,31^. In this test format, participants have to learn a series of paired stimuli (cue-target associations), and later they are presented with one of the items (cue) and have to recall the item which was previously associated with it. To investigate how memory trace strength affects retrieval related PD in such a design, Van Rijn et al.^31^ presented paired-associates to subjects and manipulated the number of tests and the amount of to-be learned associates. The authors found that the task-evoked PD in the test phase decreased by each repetition of the associates. Furthermore, they also showed that the increase in the amount of to-be-learned material led to larger task-evoked PD in the test phase. With the set size and repetition manipulation, the authors goal was to vary memory trace strength, and thus they found that increasing memory trace strength was linked to reduced PD during retrieval. Similarly, Pajkossy and Racsmány^30^ found that increasing retrieval-related PD and decreasing retention was accompanied by an increase in the amount of to-be-learned information (two, four or eight to-be-learned word pairs). According to the authors’ interpretation, the word pairs from larger set sizes were associated with weaker memory trace strength, and their retrieval was also accompanied by a higher amount of interference. These factors in turn resulted in larger PD during retrieval. These results can be interpreted in a cognitive effort framework: PD correlates with the amount of processing load during the retrieval process, which is likely the result of interference-resolution between stored items, which itself has been associated with increased PD^32^. The negative link between memory trace strength and PD evoked during cued recall is also in line with classic accounts of episodic retrieval^33,34^. In these accounts, strategic and effortful control processes are mobilized to a lesser extent during retrieval, when the retrieval cues are strongly associated with the to-be retrieved information (e.g. due to efficient encoding and less interference), because strong associates are retrieved in a reflexive manner. To sum up, in paired-associate learning tasks, memory trace strength and task-evoked PD seem to be in a negative relationship and this negative relationship might be caused by cognitive effort. Note that this is exactly the opposite pattern of what was reported in recognition memory tasks (see above).

In summary, previous research suggests that memory trace strength has differential effects on retrieval-related PDs in recognition and cued recall tasks. Specifically, memory trace strength is positively correlated with task-evoked PDs in recognition^21,23^, whereas the relationship is negative in cued recall tasks^30,31^. As outlined above, this opposing pattern may arise from the fact, that different factors mediate the link between retrieval related PD in the two forms of retrieval: in recognition, PD reflects recollection, which is positively linked to encoding quality, whereas in cued recall, PD reflects retrieval effort, which is negatively linked to encoding quality. Notably, previous studies have employed either recognition or cued recall paradigms, and to the best of our knowledge, no study has directly examined how memory trace strength influences retrieval-related PDs across recognition and cued recall using the same stimulus materials. Furthermore, previous studies investigating how memory strength affects PD during recognition and cued recall tended to use different memory strength manipulations: Whereas deep-shallow encoding manipulation was used in previous research of the pupil old/new effect^21,23^, the number of learning cycles or the learning set size was manipulated in studies investigating PDs accompanying cued recall^30,31^. Thus, the literature lacks results, which would investigate the effect of memory trace strength on retrieval-related PDs on both test formats, using the same memory strength manipulations. In the current study, we aimed to address these gaps in the literature. Specifically, we manipulated memory strength during encoding, and investigated PDs during either recognition or cued recall of the encoded items. We favored manipulating memory trace strength by varying the number of learning cycles instead of the deep-shallow processing method because pilot testing indicated floor effects for cued recall of paired associates after the shallow encoding condition. In our design, during the study phase, participants learned a series of word pairs (cue-target associations), and memory for the target items was subsequently tested using either a recognition or a cued recall task. Memory trace strength was manipulated by varying the number of presentations of each word pair, with pairs presented either once or twice. To enhance the reliance on strategic retrieval in the cued recall task, the word pairs were semantically unrelated, thereby reducing the likelihood of automatic cue-target activation. We hypothesized that this memory trace strength manipulation would affect PDs differently in the two retrieval tasks. Specifically, we predicted that increasing memory trace strength through repeated presentation of the stimuli would enhance retrieval-related PDs during successful recognition of target items. In contrast, we expected that increased memory trace strength would reduce PDs during successful cued recall of target items.

## Methods

### Participants

During the planning of our research, we were not aware of any previous study that had investigated the effects of repeated study on retrieval-related PDs. In general, previous research has demonstrated that the effects of encoding manipulations on subsequent PDs during retrieval are relatively strong for both cued recall and recognition tasks^23,30^. Based on this evidence, we estimated that the effect of repeated study would be at least medium in size; therefore, we assumed a medium effect size for both the recognition and cued recall groups. Specifically, using the software G*Power, we calculated that to detect a medium effect size (d = 0.5) with 95% power, a minimum sample size of 45 participants would be required.

To attain this minimum sample size, accounting for possible dropouts, 42 participants took part in the recognition, and 53 participants took part in the cued recall test. All participants were Hungarian undergraduate students who received monetary compensation in exchange for their participation. They gave written informed consent, and the research had been carried out in line with the Code of Ethics of the World Medical Association (Declaration of Helsinki) for experiments involving humans. The research was approved by the United Ethical Review Committee for Research in Psychology, Hungary.

The signal-to-noise ratio of trial-level pupil size data is rather low, which is generally ameliorated by examining data from multiple trials. However, in our sample, there were several participants, for whom we did have zero or only one correctly retrieved word in one of the experimental conditions. These data were considered not reliable due to high levels of noise, and because of this, we excluded every participant, who could not recall at least two items per condition in the cued recall phase. Because of this, the data of 8 participants were excluded from the final data analysis of cued recall.

After the exclusions, the final sample size was N = 45 in cued recall (female = 29, Mage = 22.40, SDage = 4.96) and N = 42 in recognition (female = 27, M age = 21.80, SD age = 2.14).

### Stimuli and materials

We selected 135 low-frequency Hungarian words. Ninety of these words were used to create 45 word pairs that served as cue-target associates during the learning phase, while the remaining 45 words functioned as foils in the recognition memory task. All words contained two or three syllables. Word pairs were constructed to avoid both semantic and phonemic relationships between the words. The same 45 cue-target pairs were used for all participants, and their assignment to strong or weak memory strength conditions was randomized.

The memory task was implemented using PsychoPy Coder^35^. Pupil size was recorded with an SMI RED500 remote eye-tracking system (SensoMotoric Instruments, Teltow, Germany). Because changes in pupil size occur relatively slowly, the original 500 Hz data were downsampled to 50 Hz, and pupil diameter was measured in millimeters. Data preprocessing was performed using custom MATLAB scripts (MathWorks, Natick, MA, USA). To control for potential luminance changes between the baseline and stimulus presentation periods, we used identical font properties and a uniform background across all trials and both test formats. Specifically, stimuli and fixation cross were presented in black Arial font (hex code: #000000) to ensure equiluminance. The height of the experimental stimuli and fixation cross was 32 pixels, and the screen background was a uniform gray (hex code: #B4B4B4).

### Procedure

The memory task comprised two phases: a learning phase and either a recognition or a cued recall phase, respectively. Specifically, participants were randomly assigned to one of two conditions: for some participants, the learning phase was followed by the recognition phase, while for others, it was followed by the cued recall phase. Thereafter, for exploratory purposes, the other memory test was also conducted (i.e. participants who started the memory testing with a recognition task, also completed the cued recall task). Because the behavior and pupil response on these secondly administered tasks could be influenced by seeing the target or the cue words during the first task, results of these secondly administered tasks are not reported or discussed in the main manuscript but are provided as supplementary material. To minimize rehearsal effects, a short retention interval was introduced between the learning and test phases, during which participants completed an arithmetic distractor task. Participants were seated in front of a screen, and eye-tracking data were recorded using a remote eye-tracker equipped with a chin rest.

The learning phase consisted of 60 trials. During each trial, word pairs were displayed at the center of the computer screen, with the cue word positioned to the left and the target word to the right of a central fixation point. Each trial began with the presentation of a fixation cross for 3 s, followed by the word pair for 5 s. To manipulate the strength of memory traces, word pairs were presented either once or twice. Specifically, 30 word pairs were presented once, while 15 word pairs were presented twice. This variation in presentation frequency was designed to create two conditions with differing levels of memory trace strength. Importantly, the experimental script pseudorandomized the presentation order of the stimuli to ensure that the two presentations of the same cue-target pair in the strong memory trace strength condition did not appear consecutively.

Instructions for memorizing the word pairs were presented on the screen prior to the learning phase. Participants were informed that, after the learning phase, they would complete both a recognition task and a cued recall task. Specifically, they were told that in the recognition task, the words originally displayed on the right side of the word pairs (i.e., the target words) would be included. For the cued recall task, participants were informed that the first element of each word pair (i.e., the cue word) would be presented on the screen, and they would need to retrieve the second, associated word (i.e., the target word). To enhance memory performance, participants were encouraged to create mental images of the word associations, as mental imagery has been shown to facilitate memory^36,37^. Following the learning phase, participants completed a three-minute distractor task involving arithmetic problems. After the distractor task, either the recognition task or the cued recall task was conducted. This was then followed by the other task (i.e. for those, who first completed the recognition task, the cued recall task was presented subsequently). Note that only the results of the firstly administered test will be presented (see above).

During the recognition task, the target words from the learning phase were presented again, randomly intermixed with an equal number of novel foils that had not been seen previously. Each word was displayed at the center of the screen, and participants were required to indicate, via a designated keypress, whether the word had been presented during the learning phase (i.e., “old”) or not (i.e., “new”). The task consisted of 90 trials (45 targets and 45 foils), and participants were required to make a decision for every trial before the presentation could proceed. Each trial began with the presentation of a fixation cross for three seconds, followed by the stimulus presentation. If participants responded within five seconds, the word remained on the screen for the remainder of the five-second interval. Thus, all words were displayed for a total of five seconds, regardless of response timing. If no keypress occurred within the five-second window, the word remained on the screen until participants made the required response, indicating either an “old” or “new” judgment.

In the cued recall task, the cue words from the learning phase were presented again on the screen, positioned to the left of the fixation cross (i.e., in the same location as during encoding). Participants were instructed to recall the associated target word and indicate, via a keypress, whether they successfully retrieved it. Each trial began with a three-second fixation period, followed by a five-second stimulus presentation interval. Participants were instructed to press a response button at the moment they experienced successful retrieval of the associate and to wait until the end of the five-second stimulus presentation interval. If a button press indicated successful retrieval, an additional response period of indefinite duration was inserted before the next fixation phase, allowing participants to verbally report the retrieved target word. Verbal responses were recorded by the experimenter and subsequently compared with the original target words; matching responses were classified as correct recalls. If participants failed to retrieve the cued item and no button press was made, the verbal response period was omitted and the next fixation period began.

### Data processing and statistical analysis

#### Behavioral data

To evaluate the effectiveness of our manipulation of memory trace strength, we analyzed reaction times for keypresses in both memory tasks. Additionally, in the cued recall task, we calculated the number of correctly recalled words for each condition. For the recognition task, we computed the percentage of hits separately for each condition.

#### Pupil size data and preprocessing

As a first step, we excluded all data samples in which the event detection algorithm of the SMI eye-tracker indicated a blink or failed to detect the pupil. On average, the proportion of missing data points due to blinks and eye detection failures was 11.20% (SD = 0.07) during the recognition task and 10.44% (SD = 0.05) during the cued recall task. For no participant did the proportion of missing data exceed the sample mean by more than three standard deviations; therefore, no participants were excluded due to low data quality. Missing segments shorter than 500 ms were linearly interpolated in accordance with established pupillometry guidelines^38^, whereas longer gaps were retained as missing values (NA)^39^. The PD time series were subsequently low-pass filtered using a second-order Savitzky–Golay filter with a frame length of 501 samples. Next, we downsampled the data from the original 500–50 Hz. We then removed data points that exceeded the mean pupil size of the specific trial by more than 3 standard deviations. These removed data points were subsequently replaced using linear interpolation.

#### Stimulus- and response-aligned time course analysis

Using the preprocessed pupil data, we created eight-second-long data segments for each trial, consisting of a three-second fixation cross period followed by a five-second cue/target word presentation. These segments were stimulus-aligned, meaning that the presentation of the cue/target word (i.e., the stimulus) always occurred at the same time point, three seconds into the segment. To mitigate the distorting effects of between-trial variability in baseline pupil size, the raw data for each trial segment underwent baseline correction^39^. Specifically, we computed the mean pupil size during the final 500 ms of the fixation cross preceding the stimulus presentation for each trial and subtracted this mean value from all data points within the segment. The baseline-corrected trial-level data segments were then stored for each participant.

Notably, memory trace strength manipulations often influence reaction times (RTs), i.e., the latency of task-related motor responses signaling a memory decision^30^. These motor responses can impact the observed PD patterns, potentially confounding how memory strength manipulations affect PD.

Specifically, differences between conditions could reflect variations in the timing of motor-related pupil size changes rather than underlying cognitive processes (i.e. differences between conditions can be caused by the difference in the latency of peak PD). To address this potential confound, we repeated the stimulus-aligned analyses using response-aligned data segments. For these, we selected 150 data points (3 s) before and after the keypress for each trial, ensuring that the behavioral response always occurred at the same time point within the segment, and so differences between the different conditions are not due to different peak latencies. Baseline correction for response-aligned data segments followed the same procedure as for stimulus-aligned data: we calculated the mean pupil size during the last 500 ms of the fixation cross preceding the stimulus presentation for each trial and subtracted this value from all data points in the response-aligned segment for that trial.

#### Statistical analysis

To investigate PD differences between conditions during recognition and cued recall, we grouped pupil responses corresponding to the two conditions (weak and strong memory trace condition) and averaged them for each participant separately. Importantly, only pupil responses accompanying correct responses were included in the analysis. We then examined the time course of the differences between conditions using these participant-level average pupil responses. First, we examined whether experimental conditions (e.g., number of presentations) differentially influenced the PD response across the two test formats: stimuli with a strong memory trace were expected to trigger an increased PD response in recognition and a decreased PD response in cued recall. Second, to replicate the pupil old/new effect, we compared the mean pupil responses for hits and correct rejections.

In each of the above analyses, we compared PD values of the different conditions for each time point using a Wilcoxon signed-rank test—that is, one comparison was made for each 20 ms during the five seconds after stimulus onset (stimulus-aligned analysis) or during the six seconds before and after the response (response-aligned analysis). To control for multiple comparisons, we used a non-parametric cluster-based permutation test using the MNE Python package^40,41^. During this analysis, data underwent 500 random permutations, whereby condition labels were shuffled for each participant during each subsequent run. For each permutation, a one-sample Wilcoxon test is conducted for each time point to assess differences between the two conditions. Significant clusters, representing continuous periods of significant difference, are identified in each permutation. As condition labels are randomly shuffled, any difference between conditions is due to random variation in the data set. Then, the sum of z-scores of the Wilcoxon tests for each cluster is calculated, and a distribution of these summed z-scores over the 500 permutations is created. In the observed data, only clusters with summed z-scores above the 95th percentile of this distribution are considered significant (corresponding to a 5% significance level).

Behavioral data (i.e., accuracy and RT) were compared using t-tests or their non-parametric counterparts, depending on whether the t-test assumptions were met.

## Results

### Behavioral results

In the recognition task, the hit rate was M = 72.14% (SD = 10.61) in the weak memory trace condition and M = 86.66% (SD = 13.73) in the strong memory trace condition. A Wilcoxon signed-rank test revealed a significant difference between the conditions, V(41) = 47, *p* < 0.001. In the cued recall task, the correct recall rate was M = 29.11% (SD = 16.73) in the weak memory trace condition and M = 51.26% (SD = 22.28) in the strong memory trace condition. As expected, a paired-samples t-test revealed a significant difference between the conditions, t(44) = − 10.05, *p* < 0.001.

We also compared RTs across the two conditions. In the recognition task, RTs were significantly faster for hits from the strong memory trace condition (M = 1.51 s, SD = 0.51) compared to hits from the weak memory trace condition (M = 1.74 s, SD = 0.38), V(41) = 152.0, *p* < 0.001. Additionally, RTs were significantly faster for hits (M = 1.66 s, SD = 0.38) compared to correct rejections (M = 1.84 s, SD = 0.48), V(41) = 176, *p* < 0.001. In the cued recall task, a similar pattern was observed: RTs were significantly faster for correctly recalled targets from the strong memory trace strength condition (M = 2.07 s, SD = 0.40) compared to correctly recalled targets from the weak memory trace strength condition (M = 2.37 s, SD = 0.56), t(44) = 3.51, *p* = 0.001.

In summary, the manipulation of memory trace strength was successful: items from the strong memory trace condition were recognized and recalled more accurately and with faster reaction times compared to items from the weak memory trace condition.

### The effect of memory trace strength on pupil dilation in cued recall

**Fig. 1:**
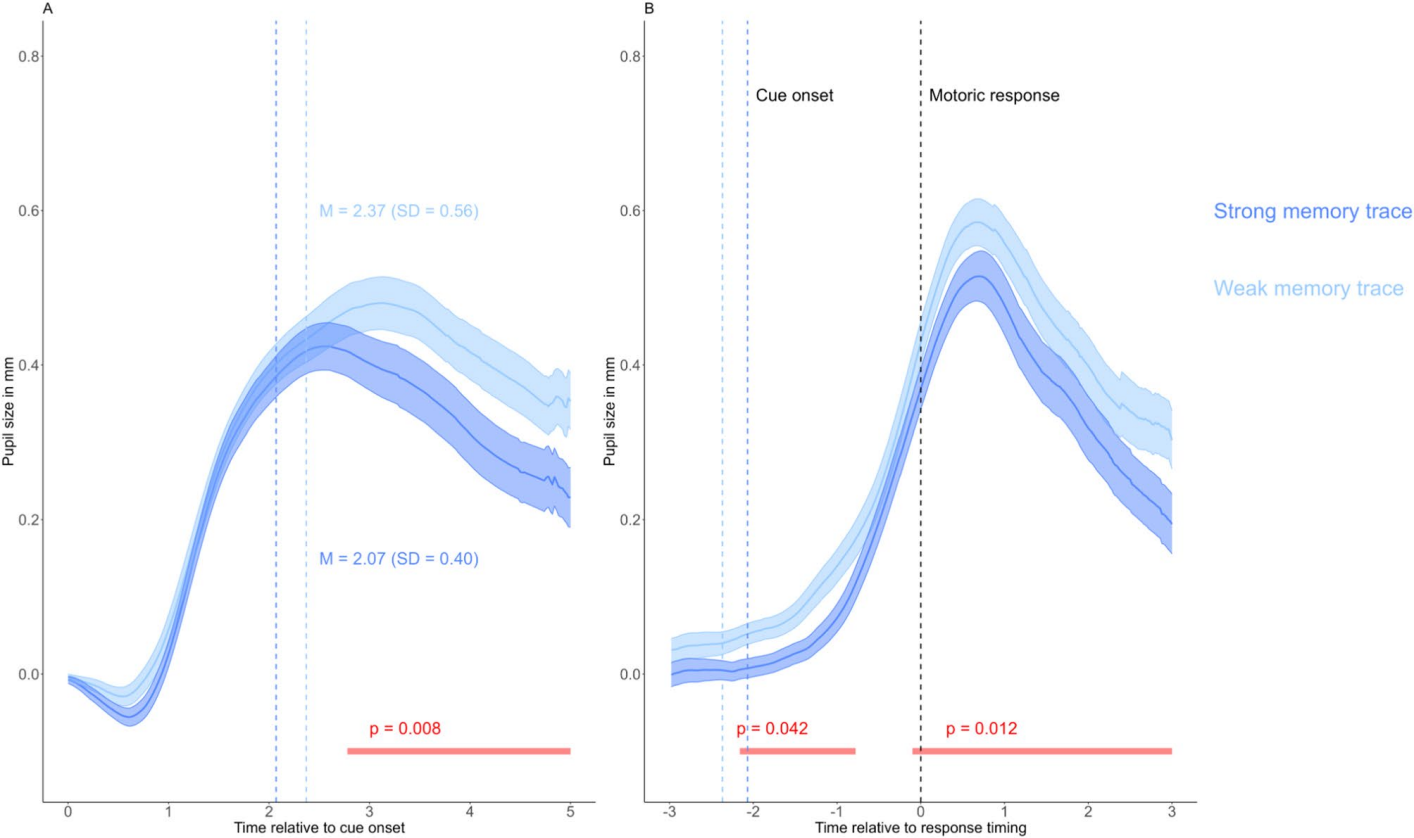
Grand-average PD curves for correct cued recall trials of different memory trace strength conditions. Note: PD differences related to memory trace strength conditions during correct cued recall trials are displayed for stimulus-aligned (**A**) and response-aligned analyses (**B**). Grand mean pupil size curves are presented, averaging the participant-level pupil size curves for both conditions separately. All values are baseline-corrected, with the mean pupil size of the 500 ms preceding stimulus onset subtracted from each data point. Significant differences between conditions during the investigated period, as revealed by cluster-based permutation testing, are indicated by red horizontal lines below the curves, accompanied by the associated p-values. Shaded areas represent the standard error of the mean. In the stimulus-aligned analysis, the colored vertical lines represent the respective response latency data for the two experimental conditions. In the response-aligned analysis, the black vertical line marks the timing of the motor response [time zero], whereas the two colored vertical lines indicate the average cue onset times relative to the motor response for the two experimental conditions. These cue-onset markers correspond to the vertical lines shown in the stimulus-aligned analysis.

The grand mean pupil size curves for both conditions are presented in Fig. 1A and B, focusing exclusively on trials where the correct target word was recalled. As shown, higher memory trace strength results in an attenuated retrieval-related PD response in both the stimulus- and response-aligned analyses. In the stimulus-aligned analysis (Fig. 1A), a non-parametric cluster-based permutation test revealed a significant cluster (*p* = 0.008) between the two conditions during the time interval from 2760 to 5000 ms relative to stimulus onset [indicated by the red horizontal line]. In the response-aligned analysis (Fig. 1B), two significant clusters were identified: one between − 2180 and − 780 ms (p = 0.042) and another between − 12 and 3000 ms (*p* = 0.012), relative to response timing, with the keypress aligned at zero.

Thus, the observed PD effects align with our hypothesis, demonstrating reduced PD during cued recall for strong cue-target associations compared to weak associations.

### The effect of memory trace strength on pupil dilation in recognition

**Fig. 2:**
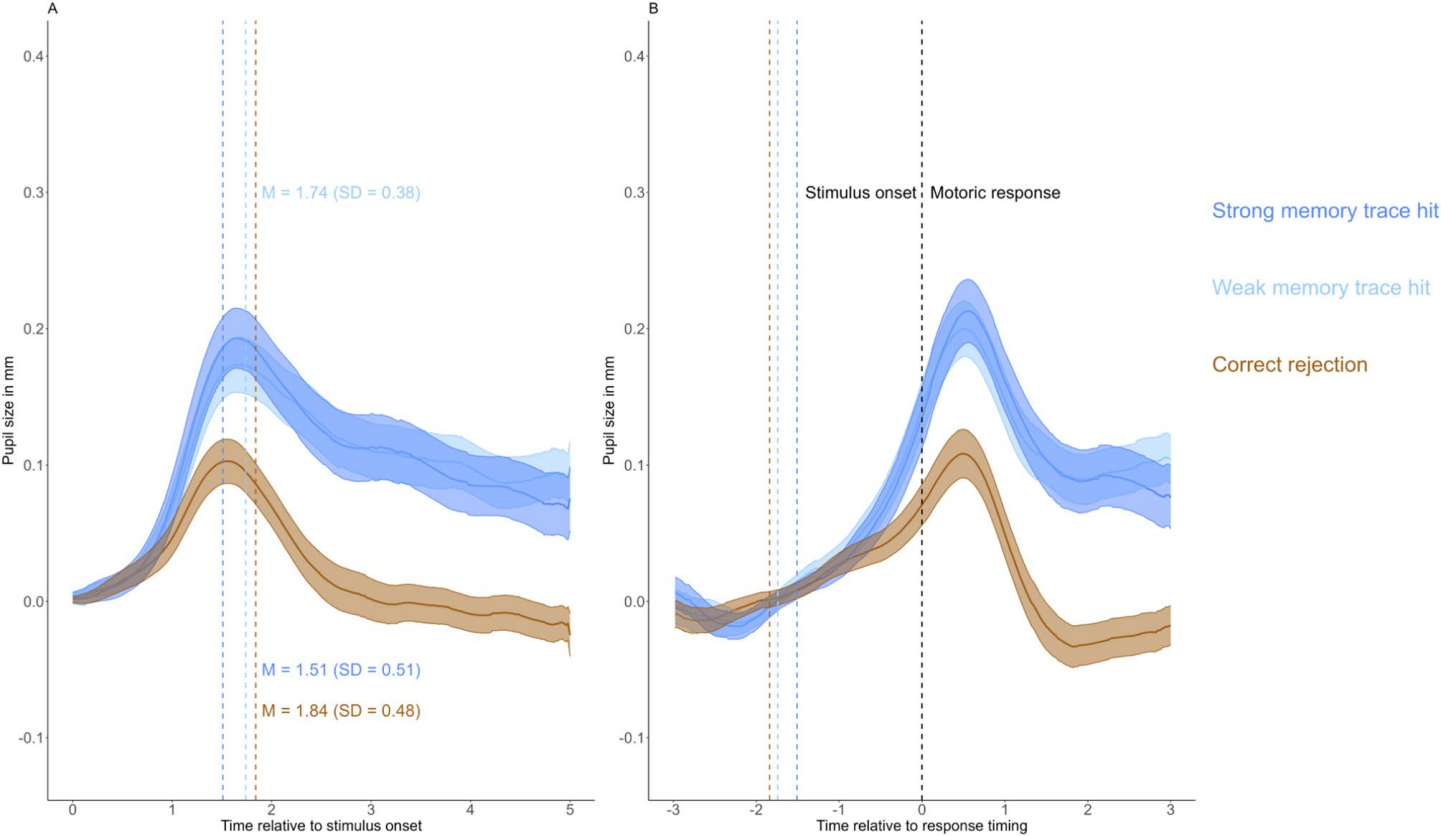
Grand-average PD curves for correctly recognized target words in both conditions and for correct rejections. Note: PD differences related to memory trace strength conditions during correct recognition trials are displayed for stimulus-aligned (**A**) and response-aligned analyses (**B**). Grand mean pupil size curves are presented, averaging the participant-level pupil size curves for the conditions separately. All values are baseline-corrected, with the mean pupil size of the 500 ms preceding stimulus onset subtracted from each data point. Shaded areas represent the standard error of the mean. In the stimulus-aligned analysis, the colored vertical lines represent the respective response latency data for the experimental conditions. In the response-aligned analysis, he black vertical line marks the timing of the motor response [time zero], whereas the three colored vertical lines indicate the average stimulus onset times relative to the motor response for the three experimental conditions. These stimulus-onset markers correspond to the vertical lines shown in the stimulus-aligned analysis.

Figure 2A and B presents the stimulus- and response-aligned grand-mean pupil size curves for correctly recognized target words in both conditions, and also for correct rejection of foil items. As can be seen, the PDs evoked by hits during the two memory trace strength conditions did not differ. This impression is also supported by the permutation test, which did not identify significant clusters between the conditions in either the stimulus-aligned (Fig. 2A) or the response-aligned (Fig. 2B) analysis. These results suggest that recognition-related PD is not modulated by memory trace strength in the same way as observed in the cued recall task.

To validate our design, we examined whether the pupil old/new effect could be replicated. As can be seen in Fig. 2, pupil response for the correctly rejected new items is smaller than for hits. In the stimulus-aligned analysis, a non-parametric cluster-based permutation test revealed a significant cluster (*p* = 0.002) between hits and correct rejection trials during the time interval from 920 to 5000 ms relative to cue onset. Similarly, in the response-aligned analysis, a significant cluster (*p* = 0.002) was identified between -640 and 3000 ms relative to response timing, with the keypress aligned at zero. In summary, we successfully replicated the effect, observing significantly increased PD during correct hit trials compared to correct rejection of novel foils.

### Recognition-related pupil dilation and memory veridicality

Our results suggest that retrieval-related PD during the recognition test is insensitive to objective measures of mnemonic trace strength. Interestingly, this finding is in line with previous findings^42,43^ suggesting that the pupil old/new effect reflects the subjective experience of a stimulus as “old,” rather than the veridicality of memory (i.e., whether the stimulus was actually presented, independent of response accuracy). Specifically, in these studies, it was shown that false alarm trials (novel foils incorrectly endorsed as “old”) elicit significantly greater PD than correct rejection trials (novel foils correctly classified as “new”). This phenomenon has been termed the subjective pupil old/new effect^43^, as these results suggest that recognition-related PD primarily tracks subjective mnemonic experience and not memory veridicality. However, it remains unclear how the memory strength manipulation applied in the present study relates to memory veridicality and subjective experience, if at all.

To address this issue, we conducted an exploratory post-hoc analysis in which we separately examined subjective mnemonic experience (“old” vs. “new” responses, independent of veridicality) and memory veridicality (whether the stimulus was actually presented, independent of the subjective experience of remembering).

**Fig. 3:**
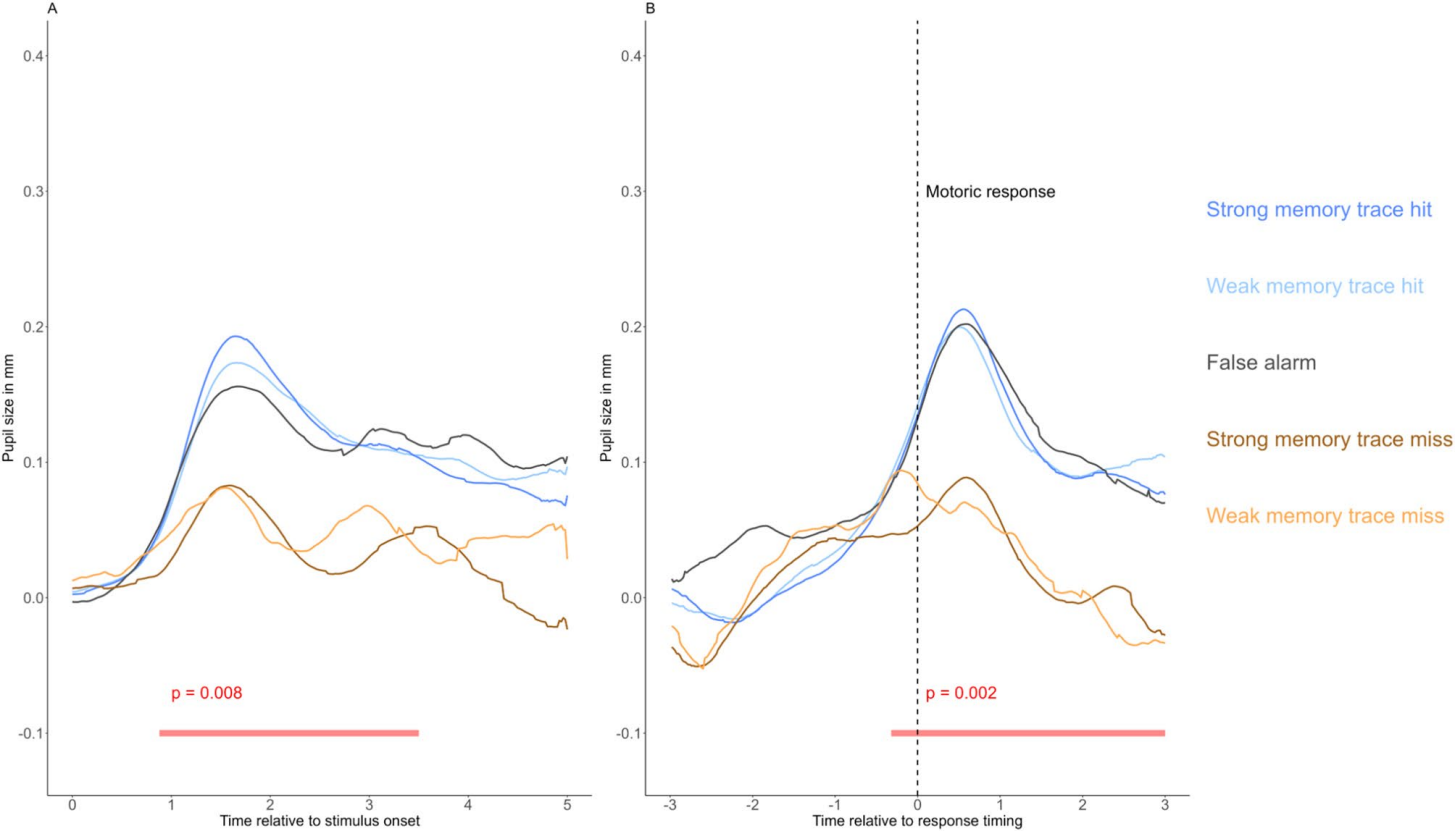
Grand-average PD curves for false alarms, misses, and hits across the two memory strength conditions. Note: Grand mean pupil size curves related to memory trace strength conditions of recognition hit trials, miss trials, and for false alarm trials are displayed for stimulus-aligned (**A**) and response-aligned analyses (**B**). Grand mean pupil size curves are presented, averaging the participant-level pupil size curves for the conditions separately. All values are baseline-corrected, with the mean pupil size of the 500 ms preceding stimulus onset subtracted from each data point. The black vertical line marks the timing of the motor response [time zero]. Significant differences between hit and miss trials, collapsed across memory trace strength conditions and identified using cluster-based permutation testing, are marked by red horizontal bars below the curves, with corresponding p-values.

On the one hand, by comparing false alarms (incorrect “old” responses to novel foils) with hit trials (correct “old” responses to studied items) across the two mnemonic trace strength conditions, subjective experience was held constant while veridicality varied. If recognition-related PD indexed a veridical memory signal independent of subjective experience, then hit trials for items presented twice should elicit greater PD than hit trials for items presented once, whereas false alarms should trigger the smallest PD. In the previous section, we already reported that no difference was found for PDs triggered by hit trials of items presented once vs. twice. In an additional analysis, we also investigated whether PD triggered by false alarms differs from hit-related PDs. Figure 3A and B presents the stimulus- and response-aligned grand-mean pupil size trajectories for false alarms, misses, and hits across the two memory strength conditions. Visual inspection reveals highly similar PD curves for hits of the two memory strength conditions and false alarms. Cluster-based permutation tests confirmed this observation, revealing no significant clusters between false alarms (gray line) and either strong-trace (deep blue line) or weak-trace hits (light blue line)—this pattern of results was consistent in both the stimulus- and response-aligned analyses. Together, these results indicate that recognition-related PD is not affected by memory veridicality, as item status (target or foil) or encoding strength did not affect PD magnitude.

On the other hand, by comparing hits (previously studied targets correctly classified as “old”) with misses (previously studied targets incorrectly classified as “new”) within each mnemonic trace strength condition, veridicality was held constant while subjective experience varied. If recognition-related PD reflects subjective mnemonic experience, then we should observe larger PD for hits than for misses. Furthermore, if PD reflects the subjective experience of recognizing a previous item, then no difference should be expected between PDs triggered by misses from the two memory strength conditions—in the case of incorrect “new” responses, encoding manipulations affecting “objective” memory strength should not influence PDs, as no recognition occurred at all, and thus no subjective feeling of recognition is present. In line with this, visual inspection of Fig. 3 revealed that miss trials exhibited comparable PD curves across mnemonic strength conditions (orange and yellow lines), again indicating a null effect of mnemonic strength manipulation. Consistent with this observation, no significant clusters were detected between strong- and weak-trace miss trials in either stimulus or response aligned data sets. In contrast, when collapsing hit and miss trials across strength conditions and directly comparing hits versus misses, significant clusters emerged from 860 to 3500 ms (*p* = 0.008) in the stimulus-aligned analysis and from − 340 to 3000 ms (*p* = 0.002) relative to response timing in the response-aligned analysis. These findings further support the interpretation that recognition-related PD reflects the subjective component of remembering rather than mnemonic veridicality.

Overall, this pattern supports a dissociation between subjective and objective components of recognition memory decisions. Crucially, recognition-related PD appears insensitive to mnemonic veridicality and, consequently, to objective indices of mnemonic trace strength. Instead, PD primarily reflects the subjective experience of remembering, suggesting that restudy-induced strengthening of mnemonic traces does not modulate this subjective component. This offers a coherent explanation for the observed null effects of mnemonic strength manipulation on recognition-related PD.

## Discussion

In our design, word pairs were presented either once or twice, followed by either a cued recall or recognition task during which retrieval-related PD was measured. Our encoding manipulation was successful, as target items presented twice during encoding were associated with higher accuracy and lower RTs in both memory tasks. Interestingly, however, despite these differences in accuracy and response speed, our encoding manipulation affected retrieval-related PDs only in the case of cued recall: Consistent with our hypotheses, recalling words that were presented twice during the learning phase elicited smaller PD compared to words encountered only once. In contrast, our predictions were not supported for the recognition task, where memory trace strength did not significantly affect the magnitude of the pupil response. The following sections will provide a more detailed interpretation of these findings.

### Memory trace strength affects PD during cued recall

Our results in the cued recall task indicate a role of cognitive effort in determining retrieval-related PD: retrieving strong memory traces evoked smaller PD. Given that cued recall relies more heavily on controlled and indirect forms of memory retrieval compared to recognition^44^, the differences in retrieval-related PD magnitude between the two conditions may reflect variations in the mental effort invested during retrieval. Retrieving target words presented only once is cognitively more demanding than retrieving words presented twice, as it involves heightened interference resolution. By repeatedly studying the word pairs, the cue-target associations are strengthened, and this can lead to reduced demand for interference resolution^45,46^, which also decreases PD^32^. When examining the temporal characteristics of the effect in Fig. 1, we can conclude that a significant difference between the two conditions emerges around the occurrence of the behavioral response. Given that pupil size lags the neurobiological activity by about 200–500 ms^47^ and that it is a summative measure of brain activity, this temporal pattern suggests that the difference is caused by the retrieval process itself and cannot be solely attributed to post-retrieval processing. Interestingly, in the response-aligned data, we also found a significant difference preceding the behavioral response (Fig. 1B). On the one hand, this might also support the notion that the observed PD effects are due to interference resolution preceding successful retrieval. On the other hand, some caution is warranted in interpreting this early effect, as the difference might be caused by RT differences: RTs for the words from the weak memory trace strength condition are larger, thus the increase of pupil size starts earlier in this condition (see horizontal lines in Fig. 1B), and this might also cause the difference. Note however, that this possible methodological constraint does not affect the interpretation of the difference following the behavioral response.

### PD triggered by correct recognition is not affected by memory trace strength

Consistent with previous studies^15,17,19–22^, we demonstrated a significant pupil old/new effect, with increased PD observed during correct “old” responses compared to correct “new” responses. Interestingly, however, the magnitude of this effect was not modulated by memory trace strength: despite the fact that words presented twice were recognized quicker and more accurately than words presented only once, correct recognition in both conditions was accompanied by similar PDs. A recent study by Ding, Whitlock, and Sahakyan^48^ reported a similar pattern with visual stimuli: repeated presentation during encoding, an approach identical to the manipulation used here, did not lead to enhanced PD during hit trials in a subsequent recognition test. Importantly, the authors also observed improved recognition accuracy (e.g. greater hit rates and lower false alarm rates) for repeated visual stimuli, thereby reinforcing and extending the conclusion that memory trace strength alone does not modulate retrieval-related PD in hit trials. Our post-hoc analysis further supports this interpretation, demonstrating that PD during the recognition test does not index memory veridicality or objective mnemonic trace strength but instead reflects the subjective experience of remembering, which appears unaffected by the implemented mnemonic trace strength manipulation (i.e., restudy).

A possible explanation for the discrepancy between the two test formats could be that retrieval-related PD during recognition reflects the recollection—familiarity dichotomy rather than memory trace strength per se^19–21,23^. If the experimental manipulation during the encoding affects the proportion of recollective responses, as is the case for the deep/shallow encoding manipulation [see^28^], then subsequently larger PD can be observed for these encoding conditions^23^. Consequently, the lack of effect for our encoding manipulation might be caused by the fact that repeated presentation during encoding did not affect the later proportion of recollective responses.

Indeed, the landmark review article by Yonelinas^28^ on the contributions of recollection and familiarity suggests that level of processing manipulations enhance recollective responses to a larger degree than responses driven by familiarity. In contrast, study duration was found to contribute to recollection and familiarity to a similar degree. Thus, our encoding manipulation might have left the proportion of recollection and familiarity-based decisions unaffected, and because the pupil old/new effect is sensitive to recollection^19–21,23^, our encoding manipulation did not affect PDs following recognition hits.

### Future directions

Our results suggest exciting opportunities for further research. In addition to pupillometry, future studies could consider incorporating microsaccadic eye movements, which are sensitive to cognitive and memory load^49^. Microsaccadic activity is modulated by cognitive load, with stronger inhibition observed under higher load^49,50^. In Kadosh et al.^50^, stronger microsaccadic suppression was also associated with better task performance, suggesting a functional link between oculomotor inhibition and memory performance. The study also showed that microsaccadic inhibition varies across encoding, maintenance, and retrieval phases^50^, suggesting that temporally resolved microsaccadic measures could be useful for probing dynamic changes in attentional demand during memory tasks.

Joint measurement of PD and microsaccades may therefore provide complementary insights into retrieval-related cognitive effort. Microsaccades co-vary with attentional demands and memory load, offering additional information about attentional dynamics during memory tasks^49,50^. Combining these measures in recall-based paradigms could help disentangle arousal and attentional components of retrieval effort and clarify how these processes interact over time.

Another important avenue for future research concerns the comparison of retrieval-related PD across different test formats, such as cued recall and recognition, under controlled encoding manipulations. As described in the introduction, previous work in cued-recall paradigms has shown that encoding manipulations that strengthen associations between study pairs, such as retrieval practice, repeated retrieval-practice blocks, or smaller set sizes, lead to reduced retrieval-related PD, consistent with lower retrieval effort^30,31,51^. By contrast, only one previous study used a similar encoding manipulation before recognition task (repeated study)^48^, whereas other recognition-memory studies have manipulated memory strength using deep-shallow encoding manipulation^21,23^. These differences highlight a critical gap: systematically comparing retrieval-related PD across test formats under identical encoding manipulations could clarify how mnemonic trace strength interacts with retrieval demands. Importantly, even manipulations that similarly enhance memory strength may do so via qualitatively distinct mechanisms (e.g., repetition vs. levels-of-processing), potentially producing divergent pupillary responses across test formats. Whereas we investigated how repeated study affects both recognition and cued-recall related PDs, future studies might use other memory strength manipulations while assessing both cued recall and recognition. The results of these studies could further elucidate the sources of divergence in retrieval-related PD, thereby providing a more comprehensive understanding of the pupillometric correlates of memory retrieval.

Finally, future investigations may benefit from examining the effects of repeated study on recognition-related PDs using designs with higher statistical power. The null effects observed in the recognition condition could, at least in part, reflect limited power to detect small effects. Accordingly, we cannot exclude the possibility that studies with larger samples might reveal a small effect of repeated study on recognition-related PDs. At the same time, the robust effect observed in the cued-recall condition may suggest that any potential effect of repeated study on recognition-related PDs is weaker than its effect on cued-recall PDs. Future research explicitly powered to detect small effects would be necessary to clarify this issue.

## Conclusion

To sum up, our findings suggest that the type of retrieval determines whether PD can be used to measure memory trace strength. Our encoding manipulations led to better accuracy and response speed in both cued recall and recognition, but this better encoding was associated with different pupil responses only in the case of cued recall, whereas we found no difference in the case of the recognition task. We propose that this pattern of results is caused by the fact that during cued recall, PD reflects retrieval effort, whereas during a recognition task, it is related to recollective recognition. Importantly, we demonstrated that the same encoding manipulation led to different patterns of pupil responses during two different forms of retrieval, which were presumably mediated by different cognitive processes. This pattern underscores that episodic memory processes must be always investigated with respect to the interaction of encoding and retrieval processes. Furthermore, it also draws attention to the fact that pupil responses are nonspecific and summative indices of brain activity, thus their interpretation must always rely on the specific information processing task which triggered them.

## Supplementary Information

Below is the link to the electronic supplementary material.

Supplementary Material 1

## Data Availability

The code for the pupil data preprocessing and the code and data for statistical analyses are publicly available on OSF, 10.17605/OSF.IO/JQZSH [https://osf.io/jqzsh/].
