# Supplementary material for: Enhancing encoding through repeated study affects retrieval related pupil dilation during cued recall, but not during recognition
Source: Sci Rep. 2026 Feb 17;16:9425. doi: 10.1038/s41598-026-40350-8 (PMC13002891; doi:10.1038/s41598-026-40350-8)
Supplement: Supplementary file 1 — Supplementary Material 1 [file 41598_2026_40350_MOESM1_ESM.docx]

**Supplementary Material**

Ádám Albi, Péter Pajkossy

As specified in the original manuscript, this supplementary document reports the results of the cued-recall and recognition tests, each of which was administered as the second test in its respective sequence. Because the pupil dilation (PD) analyses follow the same data structuring and analytical plan described in the original manuscript, only the relevant portions of the Methods and the complete Results sections are presented here.

**Methodology**

**Participants**

In this analysis we analyse the cued-recall results of the 42 participants who took the recognition test first, and recognition results of the 53 participants, who took the cued-recall test first. All participants were Hungarian undergraduate students who received monetary compensation in exchange for their participation. They gave written informed consent, and the research had been carried out in line with the Code of Ethics of the World Medical Association (Declaration of Helsinki) for experiments involving humans. The research was approved by the United Ethical Review Committee for Research in Psychology, Hungary.

The signal-to-noise ratio of trial-level pupil size data is rather low, which is generally ameliorated by examining data from multiple trials. However, in our sample, there were several participants, for whom we did have zero or only one correctly retrieved word in one of the experimental conditions. These data were considered not reliable due to high levels of noise, and because of this, we excluded every participant, who could not recall at least two items per condition in the cued recall phase. Because of this, the data of 11 participants were excluded from the final data analysis of cued recall. Regarding recognition, for two participants the software crashed after the recognition test, there no cued-recall test was administered.

After the exclusions, the final sample size was N = 31 in cued recall (female = 19,

Mage = 21.77, SDage = 2.17) and N = 51 in recognition (female = 31, M age = 22.70, SD age = 4.22).

**Pupil Size Data and Preprocessing**

On average, the proportion of missing data points due to blinks and eye detection failures was 9.25% (SD = 0.06) during the recognition task and 10.12% (SD = 0.06) during the cued recall task. For no participant did the proportion of missing data exceed the sample mean by more than three standard deviations; therefore, no participants were excluded due to low data quality.

**Results**

**Behavioral Results**

In the recognition task, the hit rate was M = 70.71% (SD = 16.98) in the weak memory trace condition and M = 85.88% (SD = 11.67) in the strong memory trace condition. A paired-samples t-test confirmed a significant difference between conditions, t(50) = -7.73, p < .001. In the cued recall task, the correct recall rate was M = 34.83% (SD = 16.75) in the weak memory trace condition and M = 56.77% (SD = 20.06) in the strong memory trace condition, with a paired-samples t-test again revealing a significant difference, t(30) = -7.53, p < .001.

Reaction times (RTs) were also compared across conditions. In the recognition task, hits from the strong memory trace condition were responded to significantly faster (M = 1.44 s, SD = 0.34) than hits from the weak memory trace condition (M = 1.56 s, SD = 0.37), t(50) = -2.95, p = .005. Additionally, hits were faster than correct rejections (M = 1.51 s, SD = 0.33 vs. M = 1.68 s, SD = 0.47), V(50) = -3.974, p < .001. In the cued recall task, RTs did not differ significantly between correctly recalled targets from the strong and weak memory trace conditions (M = 2.19 s, SD = 0.53 vs. M = 2.31 s, SD = 0.52), t(30) = -1.63, p = .11.

In summary, the memory trace strength manipulation produced the intended effect, as items from the strong memory trace condition were recognized and recalled more accurately than items from the weak condition. Regarding reaction times, items from the strong memory trace condition were recognized faster than items from the weak condition, consistent with the findings reported in the main manuscript. In the cued recall task, reaction times did not differ significantly between conditions, indicating that the speed of processing retrieval cues was comparable, despite differences in retrieval accuracy.

**The Effect of Memory Trace Strength on Pupil Dilation in Cued Recall**

The grand mean pupil size curves for both conditions are presented in Figure 1A-B, focusing exclusively on trials where the correct target word was recalled. As shown, higher memory trace strength results in an attenuated retrieval-related PD response in both the stimulus- and response-aligned analyses. However the modulating effect of mnemonic trace strength is attenuated in this analysis: in the stimulus-aligned analysis (Figure 1A), a non-parametric cluster-based permutation test revealed two non-significant clusters between the two conditions during the time interval from 280 to 520 milliseconds (p = .442) and from 3300 to 3820 milliseconds (p = .206) relative to stimulus onset. In the response-aligned analysis (Figure 1B), two significant clusters were identified: one between -1740 and -680 milliseconds (p = 0.042) and another between 1260 and 2260 milliseconds (p = 0.046), relative to response timing, with the keypress aligned at zero.

***

**Figure 1**

*Grand-average PD curves for correct cued recall trials of different memory trace strength conditions*


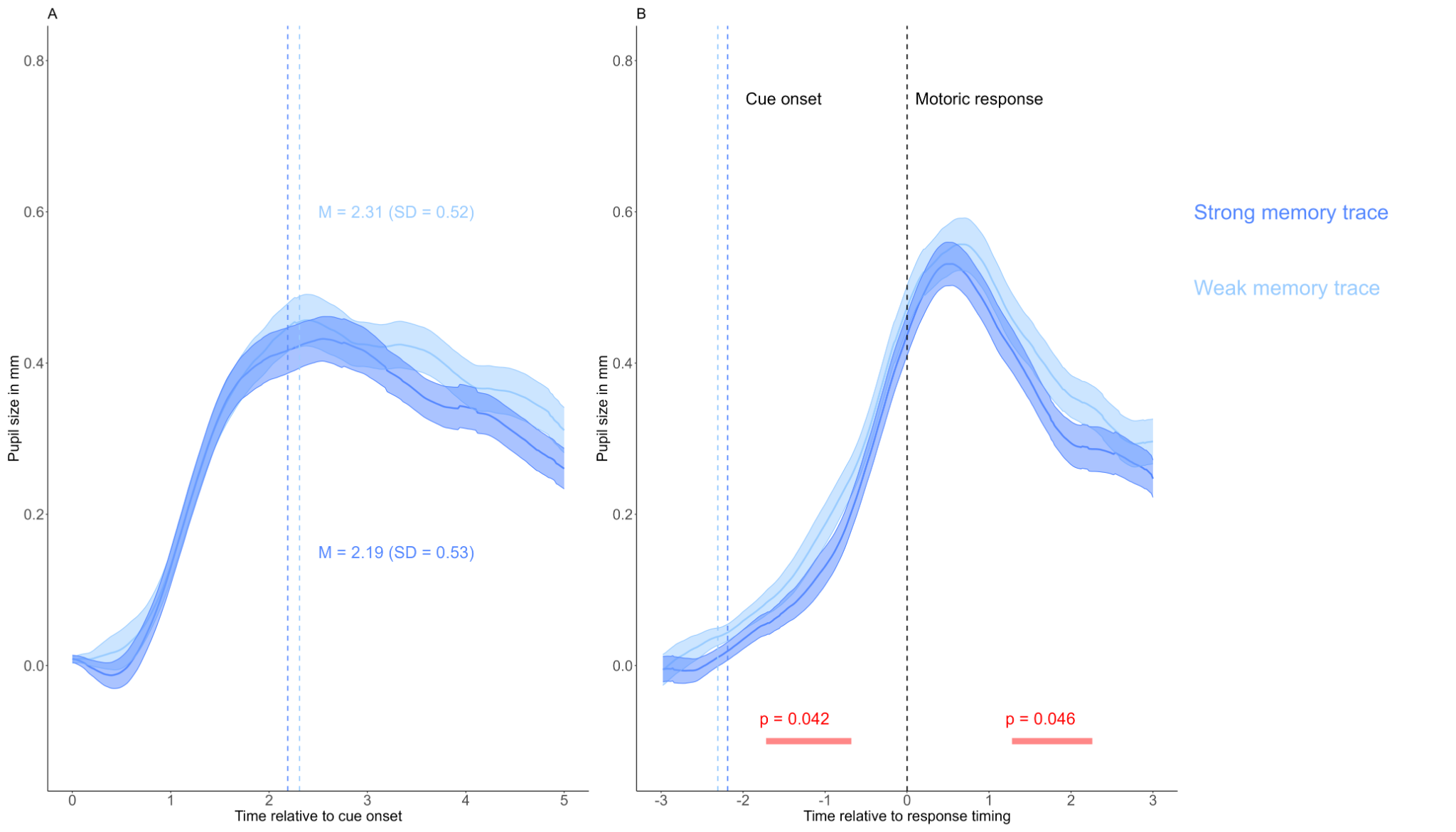


Note: PD differences related to memory trace strength conditions during correct cued recall trials are displayed for stimulus-aligned (A) and response-aligned analyses (B). Grand mean pupil size curves are presented, averaging the participant-level pupil size curves for both conditions separately. All values are baseline-corrected, with the mean pupil size of the 500 ms preceding stimulus onset subtracted from each data point. Significant differences between conditions during the investigated period, as revealed by cluster-based permutation testing, are indicated by red horizontal lines below the curves, accompanied by the associated p-values. Shaded areas represent the standard error of the mean. In the stimulus-aligned analysis, the colored vertical lines represent the respective response latency data for the two experimental conditions. In the response-aligned analysis, the black vertical line marks the timing of the motor response (time zero), whereas the two colored vertical lines indicate the average cue onset times relative to the motor response for the two experimental conditions. These cue-onset markers correspond to the vertical lines shown in the stimulus-aligned analysis.

***

In the cued recall task, the PD results were broadly consistent with the pattern reported in the main manuscript, although the effect of mnemonic trace strength was substantially attenuated. Significant effects of mnemonic trace strength emerged only in the response-aligned analysis and occurred after the peak iPD. In light of the reaction time results, which revealed no significant differences in cue-processing speed between encoding conditions, the reduced PD differences may reflect a diminished contrast in mnemonic trace strength across conditions. This attenuation likely resulted from the prior presentation of all target words during the preceding recognition task. Additionally, participant exclusions led to a markedly reduced sample size in this supplementary analysis, and the resulting decrease in statistical power may have further contributed to the weakened effects.

**The Effect of Memory Trace Strength on Pupil Dilation in Recognition**

Figure 2A–B shows the stimulus- and response-aligned grand-mean pupil size trajectories for correctly recognized target words across both memory trace strength conditions, as well as for correct rejections of foil items. As illustrated,PD associated with hit trials did not differ between the two memory trace strength conditions. This observation was corroborated by the cluster-based permutation tests, which revealed no significant clusters in the stimulus-aligned analysis (Figure 2A) and only a non-significant cluster between the conditions from -1200 to -600 ms (p = 0.13) relative to response timing in the response-aligned analysis (Figure 2B). Taken together, these results indicate that recognition-related PD is not modulated by memory trace strength, in contrast to the pattern observed in the cued recall task.

***

**Figure 2**

*Grand-average PD curves for correctly recognized target words in both conditions and for correct rejections.*


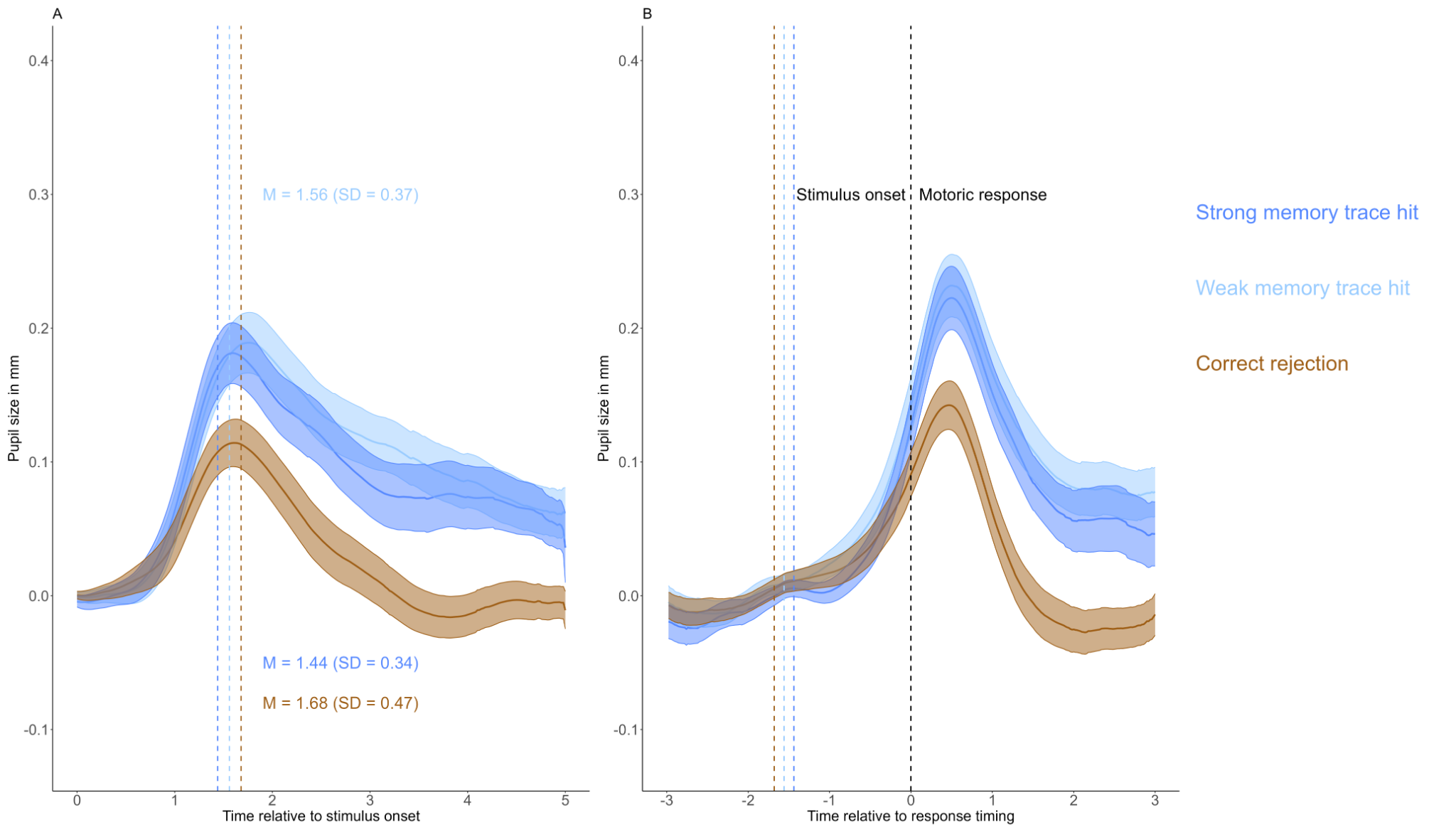


Note: PD differences related to memory trace strength conditions during correct recognition trials are displayed for stimulus-aligned (A) and response-aligned analyses (B). Grand mean pupil size curves are presented, averaging the participant-level pupil size curves for the conditions separately. All values are baseline-corrected, with the mean pupil size of the 500 ms preceding stimulus onset subtracted from each data point. Shaded areas represent the standard error of the mean. In the stimulus-aligned analysis, the colored vertical lines represent the respective response latency data for the two experimental conditions. In the response-aligned analysis, the black vertical line marks the timing of the motor response (time zero), whereas the three colored vertical lines indicate the average cue onset times relative to the motor response for the three experimental conditions. These cue-onset markers correspond to the vertical lines shown in the stimulus-aligned analysis.

*******

To validate our experimental design, we examined whether the pupil old/new effect could be replicated. As shown in Figure 2, pupil responses elicited by correctly rejected novel items were smaller than those elicited by hit trials. In the stimulus-aligned analysis, a non-parametric cluster-based permutation test revealed a significant cluster (p = 0.002) between hit and correct rejection trials in the interval from 1000 to 5000 ms relative to cue onset. Similarly, in the response-aligned analysis, a significant cluster (p = 0.002) was identified between -240 and 3000 ms relative to the response, with the keypress aligned at zero. Overall, these results indicate a successful replication of the pupil old/new effect, characterized by significantly greater PD during correct recognition of studied items compared to correct rejection of novel foils.

These results are consistent with the pattern reported in the original manuscript, as no observable effect of mnemonic trace strength was found.
